# 2,2′-[(4-Methyl-2-phenyl­imidazolidine-1,3-di­yl)bis­(methyl­ene)]diphenol

**DOI:** 10.1107/S1600536813017893

**Published:** 2013-07-03

**Authors:** Augusto Rivera, Lorena Cárdenas, Jaime Ríos-Motta, Václav Eigner, Michal Dušek

**Affiliations:** a Universidad Nacional de Colombia, Sede Bogotá, Facultad de Ciencias, Departamento de Química, Cra 30 No.45-03, Bogotá, Código Postal 111321, Colombia; b Department of Solid State Chemistry, Institute of Chemical Technology, Technická 5, 166 28 Prague, Czech Republic; c Institute of Physics AS CR, v.v.i., Na Slovance 2, 182 21 Prague 8, Czech Republic

## Abstract

The methyl-substituted imidazolidine ring of the title compound, C_24_H_26_N_2_O_2_, adopts an envelope conformation with the N atom adjacent to the methyl­ene group as the flap. The meth­yl–ethyl­ene fragment in this ring is disordered over two positions with an occupancy ratio of 0.899 (4):0.101 (4). The hy­droxy­benzyl groups are inclined at 71.57 (15) and 69.97 (19)° to the mean plane of major disorder component of the heterocyclic ring with an inter­planar angle between the two hy­droxy­benzyl groups of 66.00 (5)°. The phenyl substit­uent approaches a nearly perpendicular orientation relative to the mean plane of the imidazolidine ring, making a dihedral angle of 75.60 (12)°. This conformation is stabilized by two intra­molecular O—H⋯N bonds, which generate *S*(6) ring motifs.

## Related literature

For related structures, see: Rivera *et al.* (2012**a* ▶,b* ▶). For the synthesis of the precursor, see: Rivera *et al.* (2013 ▶). For bond-length data, see: Allen *et al.* (1987 ▶). For puckering parameters, see: Cremer & Pople (1975 ▶). For hydrogen-bond graph-set motifs, see: Bernstein *et al.* (1995 ▶).
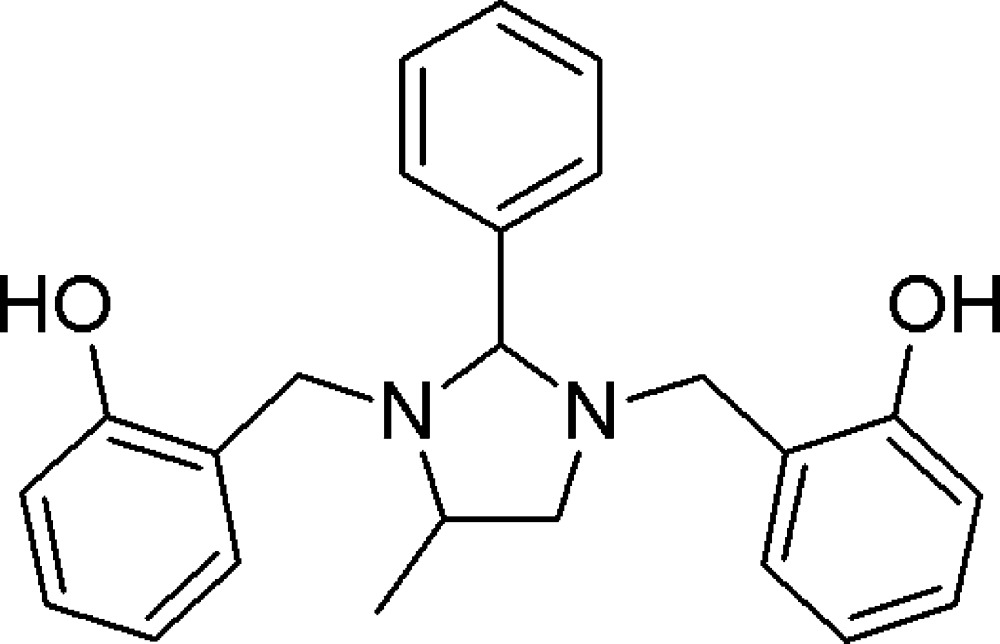


## Experimental

### 

#### Crystal data

C_24_H_26_N_2_O_2_
*M* *_r_* = 374.5
Monoclinic, 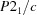

*a* = 16.8974 (8) Å
*b* = 9.4893 (5) Å
*c* = 12.5287 (6) Å
β = 92.928 (4)°
*V* = 2006.29 (17) Å^3^
*Z* = 4
Cu *K*α radiation
μ = 0.62 mm^−1^
*T* = 120 K
0.35 × 0.25 × 0.09 mm

#### Data collection

Agilent Xcalibur (Atlas, Gemini ultra) diffractometer
Absorption correction: analytical (*CrysAlis PRO*; Agilent, 2010 ▶) *T*_min_ = 0.886, *T*_max_ = 0.952
8259 measured reflections
3491 independent reflections
2704 reflections with *I* > 3σ(*I*)
*R*_int_ = 0.03

#### Refinement

*R*[*F* ^2^ > 2σ(*F* ^2^)] = 0.042
*wR*(*F* ^2^) = 0.113
*S* = 1.51
3491 reflections
276 parameters
5 restraints
H atoms treated by a mixture of independent and constrained refinement
Δρ_max_ = 0.25 e Å^−3^
Δρ_min_ = −0.18 e Å^−3^

### 

Data collection: *CrysAlis PRO* (Agilent, 2010 ▶); cell refinement: *CrysAlis PRO*; data reduction: *CrysAlis PRO*; program(s) used to solve structure: *SUPERFLIP* (Palatinus & Chapuis 2007 ▶); program(s) used to refine structure: *JANA2006* (Petříček *et al.* 2006 ▶); molecular graphics: *DIAMOND* (Brandenburg & Putz, 2005 ▶); software used to prepare material for publication: *JANA2006*.

## Supplementary Material

Crystal structure: contains datablock(s) global, I. DOI: 10.1107/S1600536813017893/sj5339sup1.cif

Structure factors: contains datablock(s) I. DOI: 10.1107/S1600536813017893/sj5339Isup2.hkl

Supplementary material file. DOI: 10.1107/S1600536813017893/sj5339Isup3.cml

Additional supplementary materials:  crystallographic information; 3D view; checkCIF report

## Figures and Tables

**Table 1:** Hydrogen-bond geometry (Å, °)

*D*—H⋯*A*	*D*—H	H⋯*A*	*D*⋯*A*	*D*—H⋯*A*
O1—H1*o*1⋯N9	0.98 (2)	1.88 (2)	2.7569 (18)	147.7 (19)
O19—H1*o*19⋯N11	1.00 (2)	1.79 (2)	2.709 (2)	152 (2)

**Fig. 1.**
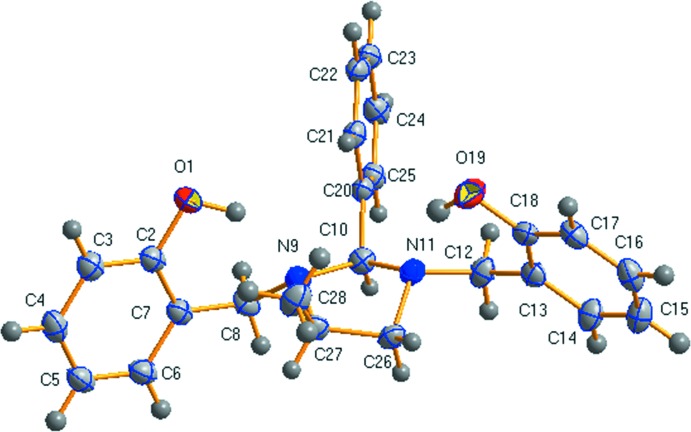
A perspective view of the title compound. Displacement ellipsoids are drawn at the 50% probability level and H atoms are shown as small spheres of arbitrary radii. Hydrogen bonds are drawn as dashed lines and only the major disorder component of the disordered section of the imidazolidine ring is shown.

## supplementary crystallographic information

### Comment

We have recently synthesized for the first time a series of tetrasubstituted imidazolidines by the reaction of *N*,*N*'-dibenzylpropane-1,2-diamine with substituted benzaldehydes, and we have provided evidence that they display intramolecular O—H···N hydrogen bonds (Rivera *et al.*, 2013). In this article, we further advance these investigations by presenting the crystal structure of the title compound.

In the title compound, C_24_H_26_N_2_O_2,_ the methyl-substituted imidazolidine ring exhibits molecular disorder over two orientations, with a refined occupancy ratio of 0.899 (4):0.101 (4) for atoms (C26, C27, C28):(C26', C27', C28'). For the major component, the imidazolidine ring (N9/C10/N11/C26/C27) adopts an envelope conformation with atom N11 as the flap: puckering parameters (Cremer & Pople, 1975) being Q2 = 0.4429 (19) Å and Φ = 75.0 (2)°. The bond lengths (Allen *et al.*, 1987) and angles are close to standard values. Within the imidazolidine ring, bond distances and angles are comparable to those found in related structures (Rivera *et al.*, 2012**a*,b*). The C2—O1 and C18—O19 bond lengths are 1.374 (2) and 1.369 (2) Å, respectively, which are comparable to other C—O bond lengths in di-Mannich bases reported by us (Rivera *et al.*, 2012**a*,b*). The hydroxybenzyl groups makes an angle of 71.57 (15)° and 69.97 (19)° with the mean plane of heterocyclic ring defined by N9,C10,C26 and C27. With reference to this plane, the phenyl ring is almost perpendicular with a dihedral angle of 75.60 (12)°. The interplanar angle between the two hydroxybenzyl groups is 66.00 (5)°.

Single-crystal X-ray diffraction analysis reveals the existence of intramolecular O—H··· N hydrogen-bonding interactions between the two N atoms of the imidazolidine ring and the hydroxyl groups in the aromatic rings with S(6) set graph motifs (Bernstein *et al.* 1995) (Table 1). However, the two observed intramolecular hydrogen bond distances were different (Table 1). It is then surprising to observe the difference in O···N distances between O1···N9 [O···N = 2.757 (2) Å] and O19···N11[O···N = 2.709 (2) Å], which are longer than those observed in a related structure (Rivera *et al.*, 2012*a*). Because the length of hydrogen bonds depends on bond strength, these results indicated that the nitrogen atoms in the title compound are weaker hydrogen bond acceptors than the nitrogen in related structure (Rivera *et al.*, 2012*a*). This fact can be explained by the presence of the aryl group at the aminal carbon, where the imidazolidine ring adopts a conformation that causes an increase in unfavorable through-space interactions between the two nitrogen lone pairs. Thus, one of the possible consequences of these structural features is the reduction in electronic density around the nitrogen atoms.

### Experimental

For the originally reported synthesis, see: Rivera *et al.* (2013). The title compound (I) were recrystallized from ethanol.

### Refinement

All hydrogen atoms were discernible in difference Fourier maps and could be refined to reasonable geometry. According to common practice H atoms bonded to C were kept in ideal positions with C–H = 0.96 Å while the coordinates of the H atoms bonded to O were refined freely. In both cases *U*_iso_(H) was set to 1.2*U*_eq_(C,*O*). All non-hydrogen atoms were refined using harmonic refinement. The disordered part of molecule was refined with bond lengths kept equal for both groups. The resulting occupancy ratio was 0.899 (4):0.101 (4).

### Crystal data

**Table:**

C_24_H_26_N_2_O_2_	*F*(000) = 800
*M**_r_* = 374.5	*D*_x_ = 1.239 Mg m^−^^3^
Monoclinic, *P*2_1_/*c*	Cu *K*α radiation, λ = 1.5418 Å
Hall symbol: -P 2ycb	Cell parameters from 3676 reflections
*a* = 16.8974 (8) Å	θ = 3.5–66.9°
*b* = 9.4893 (5) Å	µ = 0.62 mm^−^^1^
*c* = 12.5287 (6) Å	*T* = 120 K
β = 92.928 (4)°	Polygon shape, colorless
*V* = 2006.29 (17) Å^3^	0.35 × 0.25 × 0.09 mm
*Z* = 4	

### Data collection

**Table:**

Agilent Xcalibur (Atlas, Gemini ultra) diffractometer	3491 independent reflections
Radiation source: Enhance Ultra (Cu) X-ray Source	2704 reflections with *I* > 3σ(*I*)
Mirror monochromator	*R*_int_ = 0.03
Detector resolution: 10.3784 pixels mm^-1^	θ_max_ = 67.0°, θ_min_ = 5.2°
ω scans	*h* = −16→19
Absorption correction: analytical (*CrysAlis PRO*; Agilent, 2010)	*k* = −11→11
*T*_min_ = 0.886, *T*_max_ = 0.952	*l* = −14→12
8259 measured reflections	

### Refinement

**Table:**

Refinement on *F*^2^	H atoms treated by a mixture of independent and constrained refinement
*R*[*F*^2^ > 2σ(*F*^2^)] = 0.042	Weighting scheme based on measured s.u.'s *w* = 1/(σ^2^(*I*) + 0.0016*I*^2^)
*wR*(*F*^2^) = 0.113	(Δ/σ)_max_ = 0.049
*S* = 1.51	Δρ_max_ = 0.25 e Å^−^^3^
3491 reflections	Δρ_min_ = −0.18 e Å^−^^3^
276 parameters	Extinction correction: B-C type 1 Gaussian isotropic (Becker & Coppens, 1974)
5 restraints	Extinction coefficient: 3200 (500)
151 constraints	

### Fractional atomic coordinates and isotropic or equivalent isotropic displacement parameters (Å^2^)

**Table:**

	*x*	*y*	*z*	*U*_iso_*/*U*_eq_	Occ. (<1)
O1	0.10968 (7)	0.77621 (13)	0.14452 (10)	0.0335 (4)	
O19	0.40788 (9)	1.04207 (16)	0.27300 (11)	0.0460 (5)	
N11	0.28125 (8)	0.99956 (14)	0.39327 (11)	0.0262 (4)	
N9	0.16113 (8)	0.91720 (14)	0.32755 (11)	0.0262 (4)	
C3	−0.01906 (11)	0.84850 (19)	0.08234 (15)	0.0323 (5)	
C17	0.49554 (10)	1.2217 (2)	0.33593 (15)	0.0335 (6)	
C22	0.33481 (10)	0.58293 (19)	0.28115 (14)	0.0303 (5)	
C2	0.03749 (10)	0.83400 (18)	0.16509 (14)	0.0282 (5)	
C18	0.43614 (10)	1.12529 (19)	0.35565 (13)	0.0301 (5)	
C21	0.29810 (10)	0.71116 (18)	0.29487 (13)	0.0277 (5)	
C4	−0.09159 (11)	0.91012 (19)	0.10146 (15)	0.0355 (6)	
C16	0.52487 (10)	1.3093 (2)	0.41629 (15)	0.0360 (6)	
C13	0.40514 (10)	1.11650 (18)	0.45702 (13)	0.0276 (5)	
C25	0.26334 (10)	0.63967 (18)	0.46963 (13)	0.0274 (5)	
C10	0.22524 (10)	0.88368 (17)	0.40711 (13)	0.0261 (5)	
C20	0.26239 (9)	0.74138 (17)	0.39014 (12)	0.0241 (5)	
C12	0.34266 (11)	1.00921 (19)	0.47984 (13)	0.0311 (5)	
C14	0.43528 (11)	1.2068 (2)	0.53588 (14)	0.0340 (6)	
C7	0.02229 (10)	0.87866 (17)	0.26891 (13)	0.0275 (5)	
C28	0.17720 (14)	1.1089 (2)	0.19419 (16)	0.0394 (7)	0.899 (4)
C23	0.33604 (10)	0.48228 (19)	0.36133 (14)	0.0315 (5)	
C24	0.29998 (10)	0.51088 (19)	0.45540 (14)	0.0310 (5)	
C8	0.08435 (10)	0.86041 (19)	0.35794 (13)	0.0311 (5)	
C6	−0.05061 (10)	0.94177 (19)	0.28506 (15)	0.0333 (6)	
C5	−0.10736 (11)	0.95685 (19)	0.20251 (15)	0.0357 (6)	
C27	0.16255 (12)	1.07345 (19)	0.30988 (16)	0.0280 (6)	0.899 (4)
C26	0.22944 (12)	1.1238 (2)	0.38684 (17)	0.0288 (6)	0.899 (4)
C26'	0.1443 (9)	1.0661 (5)	0.3488 (14)	0.0280 (6)	0.101 (4)
C27'	0.2300 (7)	1.1160 (12)	0.3475 (10)	0.0288 (6)	0.101 (4)
C15	0.49529 (11)	1.3021 (2)	0.51673 (15)	0.0383 (6)	
H1c3	−0.008331	0.816175	0.011928	0.0387*	
H1c17	0.516222	1.227294	0.266103	0.0402*	
H1c22	0.359666	0.563345	0.21556	0.0364*	
H1c21	0.29715	0.779845	0.238562	0.0333*	
H1c4	−0.130988	0.920237	0.044006	0.0426*	
H1c16	0.566013	1.375547	0.402306	0.0432*	
H1c25	0.238354	0.658669	0.535225	0.0329*	
H1c10	0.207032	0.876559	0.478241	0.0313*	
H1c12	0.367021	0.918641	0.490924	0.0373*	
H2c12	0.31869	1.033102	0.545288	0.0373*	
H1c14	0.414141	1.203482	0.60552	0.0408*	
H1c28	0.177434	1.20937	0.185208	0.0473*	0.899 (4)
H2c28	0.227476	1.071195	0.175972	0.0473*	0.899 (4)
H3c28	0.135948	1.068532	0.148357	0.0473*	0.899 (4)
H1c23	0.361769	0.393343	0.351577	0.0378*	
H1c24	0.300297	0.441351	0.511081	0.0373*	
H1c8	0.06796	0.908229	0.420618	0.0373*	
H2c8	0.090006	0.762114	0.374792	0.0373*	
H1c6	−0.061615	0.975461	0.35501	0.04*	
H1c5	−0.157476	0.9996	0.215351	0.0429*	
H1c27	0.113089	1.118473	0.322895	0.0336*	0.899 (4)
H1c26	0.256822	1.200587	0.355095	0.0346*	0.899 (4)
H2c26	0.208953	1.141634	0.455632	0.0346*	0.899 (4)
H1c15	0.516029	1.362475	0.572938	0.046*	
C28'	0.2525 (10)	1.162 (2)	0.2369 (11)	0.042 (6)	0.101 (4)
H1o1	0.1465 (13)	0.812 (2)	0.2010 (16)	0.0402*	
H1o19	0.3584 (15)	1.002 (2)	0.3010 (17)	0.0552*	
H1c26'	0.125922	1.075665	0.419702	0.0336*	0.101 (4)
H2c26'	0.114337	1.105645	0.288888	0.0336*	0.101 (4)
H1c27'	0.237554	1.19909	0.390453	0.0346*	0.101 (4)
H1c28'	0.252753	1.082083	0.190266	0.0509*	0.101 (4)
H2c28'	0.214686	1.230137	0.208928	0.0509*	0.101 (4)
H3c28'	0.304301	1.204201	0.241575	0.0509*	0.101 (4)

### Atomic displacement parameters (Å^2^)

**Table:**

	*U*^11^	*U*^22^	*U*^33^	*U*^12^	*U*^13^	*U*^23^
O1	0.0253 (6)	0.0372 (7)	0.0383 (7)	0.0031 (5)	0.0046 (5)	−0.0046 (5)
O19	0.0470 (8)	0.0509 (9)	0.0409 (8)	−0.0076 (7)	0.0101 (7)	−0.0169 (6)
N11	0.0290 (7)	0.0204 (7)	0.0290 (7)	−0.0013 (6)	−0.0014 (6)	0.0009 (6)
N9	0.0251 (7)	0.0219 (7)	0.0314 (7)	−0.0004 (6)	0.0008 (6)	0.0031 (6)
C3	0.0309 (9)	0.0308 (9)	0.0353 (9)	−0.0024 (7)	0.0035 (7)	0.0023 (7)
C17	0.0241 (8)	0.0393 (11)	0.0373 (10)	0.0043 (8)	0.0043 (7)	0.0022 (8)
C22	0.0260 (8)	0.0305 (9)	0.0348 (9)	−0.0043 (7)	0.0045 (7)	−0.0092 (7)
C2	0.0250 (8)	0.0234 (8)	0.0366 (9)	−0.0016 (7)	0.0048 (7)	0.0012 (7)
C18	0.0269 (8)	0.0295 (9)	0.0336 (9)	0.0071 (7)	−0.0008 (7)	−0.0054 (7)
C21	0.0282 (8)	0.0271 (9)	0.0278 (8)	−0.0047 (7)	0.0003 (7)	−0.0002 (7)
C4	0.0286 (9)	0.0318 (10)	0.0455 (11)	−0.0007 (8)	−0.0045 (8)	0.0070 (8)
C16	0.0252 (9)	0.0362 (10)	0.0459 (11)	−0.0027 (8)	−0.0037 (8)	0.0094 (9)
C13	0.0283 (8)	0.0252 (9)	0.0287 (8)	0.0028 (7)	−0.0034 (7)	0.0024 (7)
C25	0.0243 (8)	0.0265 (9)	0.0315 (9)	−0.0021 (7)	0.0024 (7)	0.0013 (7)
C10	0.0285 (8)	0.0250 (9)	0.0248 (8)	−0.0010 (7)	0.0022 (7)	0.0013 (7)
C20	0.0222 (8)	0.0228 (8)	0.0271 (8)	−0.0028 (7)	−0.0003 (6)	−0.0018 (6)
C12	0.0377 (10)	0.0288 (9)	0.0263 (8)	−0.0036 (8)	−0.0031 (7)	0.0019 (7)
C14	0.0382 (10)	0.0365 (10)	0.0269 (9)	−0.0061 (8)	−0.0034 (7)	0.0017 (7)
C7	0.0244 (8)	0.0245 (9)	0.0339 (9)	−0.0008 (7)	0.0042 (7)	0.0018 (7)
C28	0.0519 (14)	0.0285 (11)	0.0372 (11)	−0.0055 (10)	−0.0041 (10)	0.0045 (9)
C23	0.0228 (8)	0.0243 (9)	0.0473 (10)	−0.0008 (7)	0.0011 (7)	−0.0053 (8)
C24	0.0272 (8)	0.0257 (9)	0.0402 (10)	−0.0012 (7)	0.0014 (7)	0.0053 (7)
C8	0.0265 (9)	0.0350 (10)	0.0322 (9)	−0.0002 (8)	0.0066 (7)	0.0009 (7)
C6	0.0278 (9)	0.0306 (10)	0.0420 (10)	0.0023 (7)	0.0069 (8)	−0.0016 (8)
C5	0.0256 (9)	0.0319 (10)	0.0499 (11)	0.0037 (8)	0.0041 (8)	0.0004 (8)
C27	0.0277 (11)	0.0219 (9)	0.0344 (13)	0.0017 (8)	0.0008 (9)	0.0021 (8)
C26	0.0352 (10)	0.0208 (9)	0.0304 (13)	0.0011 (8)	0.0013 (9)	−0.0022 (8)
C26'	0.0277 (11)	0.0219 (9)	0.0344 (13)	0.0017 (8)	0.0008 (9)	0.0021 (8)
C27'	0.0352 (10)	0.0208 (9)	0.0304 (13)	0.0011 (8)	0.0013 (9)	−0.0022 (8)
C15	0.0405 (10)	0.0361 (11)	0.0372 (10)	−0.0099 (9)	−0.0084 (8)	0.0007 (8)
C28'	0.036 (10)	0.052 (13)	0.039 (11)	−0.021 (9)	−0.002 (8)	0.016 (9)

### Geometric parameters (Å, º)

**Table:**

O1—C2	1.374 (2)	C10—H1c10	0.96
O19—C18	1.369 (2)	C12—H1c12	0.96
N11—C10	1.467 (2)	C12—H2c12	0.96
N11—C12	1.465 (2)	C14—C15	1.389 (3)
N11—C26	1.469 (2)	C14—H1c14	0.96
N11—C27'	1.500 (12)	C7—C8	1.501 (2)
N9—C10	1.469 (2)	C7—C6	1.394 (2)
N9—C8	1.473 (2)	C28—C27	1.521 (3)
N9—C27	1.500 (2)	C28—H1c28	0.96
N9—C26'	1.469 (6)	C28—H2c28	0.96
C3—C2	1.380 (2)	C28—H3c28	0.96
C3—C4	1.390 (3)	C23—C24	1.381 (3)
C3—H1c3	0.96	C23—H1c23	0.96
C17—C18	1.389 (2)	C24—H1c24	0.96
C17—C16	1.378 (3)	C8—H1c8	0.96
C17—H1c17	0.96	C8—H2c8	0.96
C22—C21	1.381 (2)	C6—C5	1.381 (3)
C22—C23	1.385 (3)	C6—H1c6	0.96
C22—H1c22	0.96	C5—H1c5	0.96
C2—C7	1.404 (2)	C27—C26	1.525 (3)
C18—C13	1.401 (2)	C27—H1c27	0.96
C21—C20	1.395 (2)	C26—H1c26	0.96
C21—H1c21	0.96	C26—H2c26	0.96
C4—C5	1.380 (3)	C26'—C27'	1.525 (19)
C4—H1c4	0.96	C26'—H1c26'	0.96
C16—C15	1.379 (3)	C26'—H2c26'	0.96
C16—H1c16	0.96	C27'—C28'	1.52 (2)
C13—C12	1.505 (2)	C27'—H1c27'	0.96
C13—C14	1.385 (2)	C15—H1c15	0.96
C25—C20	1.386 (2)	C28'—H1c28'	0.96
C25—C24	1.386 (2)	C28'—H2c28'	0.96
C25—H1c25	0.96	C28'—H3c28'	0.96
C10—C20	1.509 (2)		
			
C10—N11—C12	113.34 (13)	C2—C7—C6	117.97 (15)
C10—N11—C26	102.83 (13)	C8—C7—C6	122.06 (16)
C10—N11—C27'	103.5 (5)	C27—C28—H1c28	109.47
C12—N11—C26	112.84 (13)	C27—C28—H2c28	109.47
C12—N11—C27'	127.6 (5)	C27—C28—H3c28	109.47
C10—N9—C8	111.98 (13)	H1c28—C28—H2c28	109.47
C10—N9—C27	107.30 (13)	H1c28—C28—H3c28	109.47
C10—N9—C26'	103.2 (6)	H2c28—C28—H3c28	109.47
C8—N9—C27	114.89 (14)	C22—C23—C24	119.49 (16)
C8—N9—C26'	97.1 (6)	C22—C23—H1c23	120.25
C2—C3—C4	119.68 (17)	C24—C23—H1c23	120.25
C2—C3—H1c3	120.16	C25—C24—C23	120.18 (16)
C4—C3—H1c3	120.16	C25—C24—H1c24	119.91
C18—C17—C16	120.22 (17)	C23—C24—H1c24	119.91
C18—C17—H1c17	119.89	N9—C8—C7	110.91 (14)
C16—C17—H1c17	119.89	N9—C8—H1c8	109.47
C21—C22—C23	120.50 (16)	N9—C8—H2c8	109.47
C21—C22—H1c22	119.75	C7—C8—H1c8	109.47
C23—C22—H1c22	119.75	C7—C8—H2c8	109.47
O1—C2—C3	119.08 (15)	H1c8—C8—H2c8	108
O1—C2—C7	120.03 (14)	C7—C6—C5	121.38 (17)
C3—C2—C7	120.89 (16)	C7—C6—H1c6	119.31
O19—C18—C17	118.21 (16)	C5—C6—H1c6	119.31
O19—C18—C13	121.29 (15)	C4—C5—C6	119.63 (17)
C17—C18—C13	120.49 (16)	C4—C5—H1c5	120.19
C22—C21—C20	120.31 (15)	C6—C5—H1c5	120.19
C22—C21—H1c21	119.85	N9—C27—C28	111.35 (15)
C20—C21—H1c21	119.85	N9—C27—C26	103.51 (14)
C3—C4—C5	120.43 (17)	N9—C27—H1c27	113.22
C3—C4—H1c4	119.78	C28—C27—C26	112.46 (17)
C5—C4—H1c4	119.78	C28—C27—H1c27	104.42
C17—C16—C15	120.21 (17)	C26—C27—H1c27	112.13
C17—C16—H1c16	119.9	N11—C26—C27	101.74 (14)
C15—C16—H1c16	119.9	N11—C26—H1c26	109.47
C18—C13—C12	120.74 (15)	N11—C26—H2c26	109.47
C18—C13—C14	117.97 (16)	C27—C26—H1c26	109.47
C12—C13—C14	121.27 (15)	C27—C26—H2c26	109.47
C20—C25—C24	120.70 (16)	H1c26—C26—H2c26	116.23
C20—C25—H1c25	119.65	N9—C26'—H1c26'	109.47
C24—C25—H1c25	119.65	N9—C26'—H2c26'	109.47
N11—C10—N9	102.44 (12)	C27'—C26'—H1c26'	109.47
N11—C10—C20	112.26 (13)	C27'—C26'—H2c26'	109.47
N11—C10—H1c10	113.69	H1c26'—C26'—H2c26'	120.28
N9—C10—C20	113.32 (13)	N11—C27'—C26'	107.3 (8)
N9—C10—H1c10	112.64	N11—C27'—C28'	113.3 (11)
C20—C10—H1c10	102.9	N11—C27'—H1c27'	109.44
C21—C20—C25	118.82 (15)	C26'—C27'—C28'	112.5 (12)
C21—C20—C10	120.40 (14)	C26'—C27'—H1c27'	110.35
C25—C20—C10	120.77 (14)	C28'—C27'—H1c27'	103.96
N11—C12—C13	112.27 (13)	C16—C15—C14	119.45 (17)
N11—C12—H1c12	109.47	C16—C15—H1c15	120.27
N11—C12—H2c12	109.47	C14—C15—H1c15	120.27
C13—C12—H1c12	109.47	C27'—C28'—H1c28'	109.47
C13—C12—H2c12	109.47	C27'—C28'—H2c28'	109.47
H1c12—C12—H2c12	106.52	C27'—C28'—H3c28'	109.47
C13—C14—C15	121.65 (17)	H1c28'—C28'—H2c28'	109.47
C13—C14—H1c14	119.18	H1c28'—C28'—H3c28'	109.47
C15—C14—H1c14	119.17	H2c28'—C28'—H3c28'	109.47
C2—C7—C8	119.96 (15)		

### Hydrogen-bond geometry (Å, º)

**Table:**

*D*—H···*A*	*D*—H	H···*A*	*D*···*A*	*D*—H···*A*
O1—H1*o*1···N9	0.98 (2)	1.88 (2)	2.7569 (18)	147.7 (19)
O19—H1*o*19···N11	1.00 (2)	1.79 (2)	2.709 (2)	152 (2)
O1—H1*o*1···C8	0.98 (2)	2.32 (2)	2.844 (2)	112.5 (16)
O19—H1*o*19···C12	1.00 (2)	2.27 (2)	2.884 (2)	118.6 (16)
O19—H1*o*19···C28′	1.00 (2)	2.45 (3)	2.877 (18)	105.1 (16)
C26′—H2*c*26′···C28	0.96	1.63	2.082 (17)	103.83
C28′—H3*c*28′···O19	0.96	2.35	2.877 (18)	114.12
C26—H1*c*26···C28′	0.96	1.52	1.973 (15)	102.84
